# A Depth-Based Weighted Point Cloud Registration for Indoor Scene

**DOI:** 10.3390/s18113608

**Published:** 2018-10-24

**Authors:** Shuntao Liu, Dedong Gao, Peng Wang, Xifeng Guo, Jing Xu, Du-Xin Liu

**Affiliations:** 1 AVIC Chengdu Aircraft Industrial (Group) Co., Ltd., Chengdu 610092, China; liushuntao99@tsinghua.org.cn; 2 Department of Mechanical Engineering, Qinghai University, Xining 810016, China; gaodd@qhu.edu.cn (D.G.); p-wang15@tsinghua.org.cn (P.W.); 3 State Key Laboratory of Tribology and Department of Mechanical Engineering, Tsinghua University, Beijing 100084, China; guoxifengabc@163.com (X.G.); jingxu@mail.tsinghua.edu.cn (J.X.)

**Keywords:** point cloud registration, iterative closest point, depth measurement error

## Abstract

Point cloud registration plays a key role in three-dimensional scene reconstruction, and determines the effect of reconstruction. The iterative closest point algorithm is widely used for point cloud registration. To improve the accuracy of point cloud registration and the convergence speed of registration error, point pairs with smaller Euclidean distances are used as the points to be registered, and the depth measurement error model and weight function are analyzed. The measurement error is taken into account in the registration process. The experimental results of different indoor scenes demonstrate that the proposed method effectively improves the registration accuracy and the convergence speed of registration error.

## 1. Introduction

To obtain a complete scene point cloud map, it is necessary to measure the point cloud from different positions or perspectives several times, and each measurement result is in different coordinate systems. Therefore, the coordinate system transformation should be carried out for the point clouds measured at different positions, and the same point in the point cloud measured at different positions should be transformed to the same position [1,2]. This process is called point cloud registration. For the map reconstruction of point clouds in static scenes, the coordinate transformation between point clouds can be considered as rigid transformation. Point cloud registration can be generally divided into two processes: rough registration and fine registration. Rough registration can provide initial values of optimization for fine registration. While using the point cloud reconstruction device with motion estimation functions such as inertial measurement unit or rotating table, the relative motion between point clouds can be obtained directly, so it is easy to coarse registration of point cloud. Otherwise, the transformation matrix between two point clouds should be calculated.

To reduce the influence of measurement errors on registration accuracy, a novel point cloud registration method is proposed in this paper. Firstly, the transfinite and interference points in two point clouds are filtered by preprocess. The matching relations of points between two point clouds are found by iterative closest point method. To improve the registration accuracy and speed, the point pairs with smaller Euclidean distances are used for registration, and the weights of each point pair are assigned according to the depth values. Finally, the rotation and translation matrices are calculated and the registration errors are obtained. The depth camera is used to collect environmental point clouds, and the experimental results show that the proposed method can effectively improve the registration accuracy and speed.

The major contributions of this study are as follows:

(1) during the iterative registration process, only the point pairs with smaller Euclidean distances are used, which improves the accuracy of registration;
(2) by incorporating the measurement error into the registration process, the weights are assigned to each point pair according to the measurement depth error and the convergence speed of registration error is improved.

The rest of this paper is organized as follows. Section 2 discusses the related work. The notations, assumptions and problem definition are given in Section 3. Section 4 presents the novel registration method for point clouds. We provide the experimental results and analysis in Section 5, and conclude the paper and discuss the future work in Section 6.

## 2. Related Work

On rough registration based on two-dimensional features, Lin et al. [3] proposed an efficient three-dimensional registration method based on a two-dimensional local feature matching, which is demonstrated that the method is robust to a larger difference of viewing angle. Many three-dimensional feature points can also be used for registration, such as Fast Point Feature Histograms (FPFH) features [4] and local three-dimensional feature. Yang et al. [5] proposed a registration method to provide marker-free and multi-view registration based on the semantic feature points extracted. This method mainly took advantage of the limited position and attitude of the sensor (only moving on the horizontal plane and rotating in gravity direction), and it was suitable for large-scale outdoor scene such as Terrestrial Laser Scanning (TLS). However, it needed to establish point cloud grids during the point cloud segmentation, which would increase the amount of calculation.

At present, the point cloud fine registration method used widely is the Iterative Closest Point (ICP) algorithm which was proposed by Besl et al. [6]. The basic principle is to iteratively calculate the nearest points between each point in the registration points and the fixed reference point, and the two nearest points are as matching points. The ICP algorithm can be used for registration of broken line points, curve points, surface points, dense points and triangular points. It is widely used in point cloud registration because it does not need the matching relationship between point sets and also has a faster calculation speed. However, the ICP algorithm also has the following shortcomings: the point clouds to be registered should have a large overlap area; easy to fall into local optimum; high requirements for initial values. For this reason, many variant ICP algorithms based on measurement scene and sensor type were proposed [7]. Chen et al. [8] presented an approach that works on range data directly and registers successive views with enough overlapping area to get an accurate transformation between views. It was performed by minimizing a functional that did not require point-to-point matches. Segal et al. [9] proposed a generalization of the ICP algorithm which takes into account the locally planar structure of both scans in a probabilistic model.

Another type of method for fine registration is based on probability distribution. For example, the registration method based on normal distribution transformation is not to establish the matching relationship between point clouds, but to divide the point clouds into blocks, and then use the probability distribution of these point clouds to represent the point clouds. The matching problem is transformed into the expectation maximization problem, which is used to deal with sparse point clouds from laser radar. Agamennoni et al. [10] presented a point cloud registration method based on probability distribution. Unlike the “point to point” matching registration, the method was a weighted matching registration. The transformation matrix was estimated by expectation maximization, and the accuracy of registration is significantly improved.

We proposed a point-based rigid registration method which provided different weights for different locations and directions of the measured points based on the measurement error distribution properties of laser tracker [11]. The experimental results showed that the proposed method clearly performs better for the same fiducial point number and distribution than the traditional method.

After rough and fine registrations, two point clouds are transformed into the same coordinate. Due to the accumulated error, it is necessary to do global registration and optimization for registered point clouds. In the present achievements, the influence of measurement errors on registration is seldom considered in the field of visual measurements. Because the point cloud registration is the basis of constructing a complete three-dimensional point cloud map or other subsequent processing, it is very important to consider the measurement error of point cloud in the registration process.

## 3. Problem Definition

The goal of this paper is to improve the ICP algorithm by using point pairs with smaller Euclidean distances for registration. At the same time, the measurement error is considered in the registration process, and the weights are assigned to each point pair according to the measurement error. The key of this paper is to establish the measurement error model

(1) e=ϵ(x,y,z,p)

where (x,y,z) represents the coordinate value of point and p contains the intrinsic parameters of depth camera. Based on the error *e*, the weight function affecting registration is constructed

(2) w=ω(e)

The amount of point pairs with smaller Euclidean distances for registration is notated as *Q*. The rotary and translation matrices can be calculated by

(3) R,T=f(Y,X,Q,w)

where X and Y are the source and target point clouds.

To focus more on verifying the feasibility of the proposed method, this study only considers the point cloud pre-processing and registration process, and makes the following assumptions:

(1) the source and target point clouds are obtained by depth camera from two different static postures;
(2) there is a large overlap area between the source and target point clouds.

The object of this paper is to determine the functions ϵ(·), ω(·), and f(·) under assumed conditions, and verify the method by experiments.

## 4. Method

### 4.1. Principle of Point Cloud Registration

In three-dimensional reconstruction, it is necessary to change the position and attitude of the camera for multiple scene acquisition, and then get the whole three-dimensional point cloud of the target scene by point cloud registration and fusion. Therefore, each point cloud is measured in different local coordinate systems. The main goal of this paper is to improve the registration accuracy of two point clouds.

**Figure 1:**
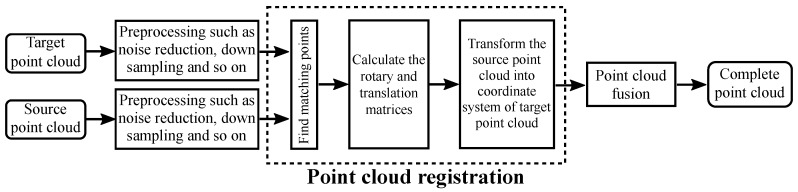
The flow chart of point clouds registration.

The origin of the local coordinate system is located at the center of the camera, which is notated as $o_{c}x_{c}y_{c}z_{c}$. Point cloud registration is to transform point clouds in different coordinate systems into the same coordinate system, and ensure that the same point in different point clouds can coincide as much as possible after registration. The flow chart of point clouds registration is shown in Figure 1.

The goal of point cloud registration is to transform local point clouds into the global coordinate system.

(4) $${\left\{X^{\prime }\right\}}_{g}=H_{g}^{c}{\left\{X\right\}}_{c}$$

where ${\left\{X\right\}}_{c}={\left\{X_{1},X_{2},\ldots ,X_{N}\right\}}_{c}$ represents the source point cloud in local coordinate system with *N* points to be registered, ${\left\{X^{\prime }\right\}}_{g}$ is the registered point cloud in global coordinate system. The target point cloud to be registered can be either a registered point cloud or a point cloud fused by the previous measurements in global coordinate system.

The target point cloud is notated as {Y} and the source point cloud is notated as {X}. The key to registration is to calculate the transformation matrix $H_{g}^{c}$, and minimize the error function

(5) $$\epsilon_{r}=\sum_{i=1}^{N}{∥Y_{i}-H_{g}^{c}X_{i}∥}^{2}$$

where $Y_{i}$ is one point of target point cloud in global coordinate system which is matched to $X_{i}$.

For point cloud registration in static scenes, it can be considered that different point clouds satisfy the rigid transformation. Therefore, the Formula (5) can be written as

(6) $$\epsilon_{r}=\sum_{i=1}^{N}{∥Y_{i}-R_{g}^{c}X_{i}-T_{g}^{c}∥}^{2}$$

where $R_{g}^{c}$ and $T_{g}^{c}$ are the rotation and translation matrices of rigid body transformation.

There are two main issues in solving Formula (6): (1) finding the point $Y_{i}$ in target point cloud $\left\{Y_{i}\right\}$ which matches the point $X_{i}$; (2) optimization solution.

Assuming that a matching relationship has been found, it can be solved as follows:

(1) calculate the centers of target point cloud and point cloud to be registered
  (7) $$\begin{array}{c} \bar{X}=\frac{1}{N}\sum_{i=1}^{N}X_{i}\\ \bar{Y}=\frac{1}{N}\sum_{i=1}^{N}Y_{i} \end{array}$$
(2) calculate the covariance matrix
  (8) $$\Sigma =\frac{1}{N}\left[\left(Y-\bar{Y}\right){\left(X-\bar{X}\right)}^{T}\right]$$
(3) construct symmetric matrix
  (9) $$Q\left(\Sigma \right)=\left[\begin{array}{cc} tr(\Sigma ) & \Delta^{T}\\ \Delta & \Sigma +\Sigma^{T}-tr\left(\Sigma \right)I_{3\times 3} \end{array}\right]$$
  where $\Delta =[{\left(\Sigma -\Sigma^{T}\right)}_{23},{\left(\Sigma -\Sigma^{T}\right)}_{31},{\left(\Sigma -\Sigma^{T}\right)}_{12}]$
(4) perform eigenvalue decomposition of matrix Q(Σ), and the quaternion $v_{g}^{c}$ corresponding to the rotation matrix $R_{g}^{c}$ is obtained by taking the eigenvector corresponding to the maximum eigenvalue.
  (10) $$v_{g}^{c}=Q\left(R_{g}^{c}\right)$$

The translation matrix is

(11) $$T=\bar{Y}-R_{g}^{c}\bar{X}$$

### 4.2. Depth Measurement Error Model

**Figure 2:**
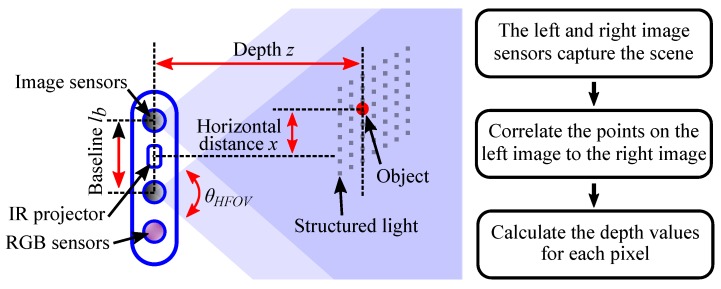
The depth perception based on active infrared stereo vision technology.

To improve the 3D point cloud modeling of complex indoor environment, a novel point cloud registration method based on Intel${}^{®}$ RealSense${}^{\mathrm{TM}}$ depth camera is proposed in this paper, which can reduce the influence of measuring errors. The Intel${}^{®}$ RealSense${}^{\mathrm{TM}}$ depth camera adopts active infrared (IR) stereo vision technology, which is shown in Figure 2. The depth perception based on stereo vision is implemented by two image sensors and an infrared projector. The infrared projector projects non-visible structured IR pattern to improve depth accuracy in scenes. The depth calculation process in presented in the right of Figure 2. The depth image processor obtains the scene data by the two image sensors, and the depth values for each pixel can be calculated by correlating the points on the left image to the right image.

In fact, there will be measurement error in depth camera. If the measurement error is not considered, the original error will be accumulated and enlarged when the depth map or point cloud is applied, resulting in poor results.

To analyse and calculate the depth error, the symbols for camera parameters are defined as follows: the depth between the origin of camera and the object is *z*, the size of subpixel is $p_{s}$, the focal length is $l_{f}$, and the baseline of two cameras is $l_{b}$. The horizontal resolution is $r_{X}$, and the horizontal field of view is $\theta_{HFOV}$. The RMS error represents the depth noise for a localized plane fit to the depth values and is defined as [12]

(12) $$e=\frac{z^{2}p_{s}}{l_{f}l_{b}}$$

where the focal length is

(13) $$l_{f}=\frac{r_{X}}{2\tan(\frac{\theta_{HFOV}}{2})}$$

It can be concluded that the depth error is proportional to the square of the depth value.

As described in Section 4.1, in general ICP algorithms, the weights of different pairs of points are equal when the registration error is minimized. In fact, as the depth increases, the measurement error will increase and the credibility of measurement result will also change. To quantify the impact of errors on measurement results, the scalar η of relative error is defined and simplified as

(14) $$\eta =\frac{e}{z}=z\times \frac{2p_{s}\tan\left(\frac{\theta_{HFOV}}{2}\right)}{r_{X}l_{b}}$$

When the point cloud registration error is minimized, the weights of different point pairs are defined as *w*

(15) $$w=\frac{1}{\eta }$$

### 4.3. Depth-Based Weighted ICP Registration

Section 4.1 assumes that the matching relations of two point clouds to be registered are known, and the transformation matrix can be calculated. However, for two point clouds with large amount of points, it is difficult to find point pairs between them. The classic ICP algorithm is to calculate rotation and translation matrices of two point clouds by finding closest point pairs iteratively.

During iterative closest points, all the points in source point cloud are used to match points in target point cloud. Then the distances between point $X_{i}$ in source point cloud and all points {Y} in target point cloud are calculated. The point corresponding to minimum distance (nearest neighbor) is taken as the matching point of $X_{i}$.

(16) $${\dot{Y}}_{i}=\underset{Y_{i}\in \left\{Y\right\}}{argmin}{∥Y_{i}-X_{i}∥}^{2}$$

Using Formula (16), a new target point cloud $\left\{\dot{Y}\right\}$ that matches all the points in the source cloud can be obtained. In fact, the two point clouds obtained in different positions and attitudes cannot completely coincide. For more accurate registration, we choose Q(Q<N) point pairs with smaller Euclidean distances as the object point clouds to calculate the transformation matrix and registration error. The *Q* is generally the number of overlap points of two point clouds. The number is difficult to be calculated accurately, so it can use an approximate value.

(17) $$\begin{array}{cc} X^{\prime } & =\underset{Q}{argmin}\left\{X\right\}\\ Y^{\prime } & =\underset{Q}{argmin}\left\{\dot{Y}\right\} \end{array}$$

Based on new target and source point clouds $Y^{\prime }$ and $X^{\prime }$, the transformation matrix $H_{g}^{c}$ can be calculated by Formulas (7)–(11).

(18) $$H_{g}^{c}=argmin\;\epsilon_{r}$$

The coordinate transformation for source point cloud is performed by Formula (4), and the registration error is calculated by Formula (6). If the registration error changes less than the pre-set threshold $\epsilon_{TH}$ or the number of iterations is greater than the pre-set threshold $N_{TH}$, the algorithm terminates; otherwise the iteration will continue.

The above is the procedure of general ICP algorithm, and the weights of different point pairs are equal while minimizing the registration error. To reduce the influence of measurement errors on point cloud registration results, the depth error should be incorporated into the registration process by weighting. Therefore, different weights can be assigned to different point pairs according to the depth errors, and the registration accuracy can be improved during the least square optimization process. The error function can be rewritten as follows:

(19) $$\epsilon =\sum_{i=1}^{Q}w_{i}{∥Y_{i}^{\prime }-R_{g}^{c}X_{i}^{\prime }-T_{g}^{c}∥}^{2}=\sum_{i=1}^{Q}\frac{1}{z\times \frac{2p_{s}\tan\left(\frac{\theta_{HFOV}}{2}\right)}{r_{X}l_{b}}}{∥Y_{i}^{\prime }-R_{g}^{c}X_{i}^{\prime }-T_{g}^{c}∥}^{2}$$

By continuously minimizing the objective error function ϵ, the optimal rotation and translation matrices can be obtained to improve the registration accuracy of point clouds.

## 5. Experiments and Results

To improve the accuracy of point cloud registration in complex indoor scenes, this paper proposed a novel point cloud registration method by reducing the number of point pairs and incorporating the measurement errors into the registration process. The Section 4 presents the detailed depth error model and registration method. This section will verify the effectiveness of the proposed method through multiple scene registration experiments and result analysis.

### 5.1. Depth Camera

**Figure 3:**
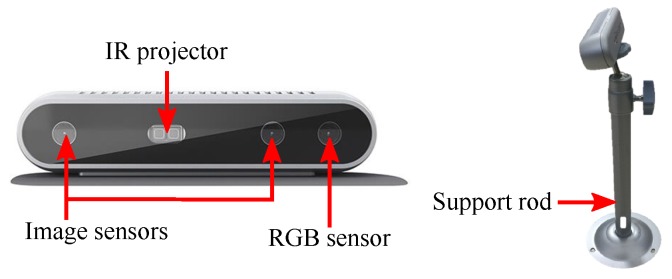
Intel${}^{®}$ RealSense${}^{\mathrm{TM}}$ D415 depth camera.

In this experiment, the point cloud data is obtained by Intel${}^{®}$ RealSense${}^{\mathrm{TM}}$ D415 depth camera. As shown in Section 4.2, the depth camera adopts active infrared stereo vision technology to measure the depth of the environment. It consists of two image sensors, an IR projector, and a RGB sensor, which is shown in Figure 3. The key specifications of depth camera is presented in Table 1.

To verify the effectiveness of proposed method, the parameters of the depth camera are determined according to the experimental requirements: $l_{b}=55$ mm, $\theta_{HFOV}=65^{\circ }$, $r_{X}=1280$, $p_{s}=0.08$. Therefore, the depth error *e* and scalar η are calculated as follows:

(20) $$\begin{array}{cc} e & =0.0014479\times z^{2}\\ \eta & =0.0014479\times z \end{array}$$

**Figure 4:**
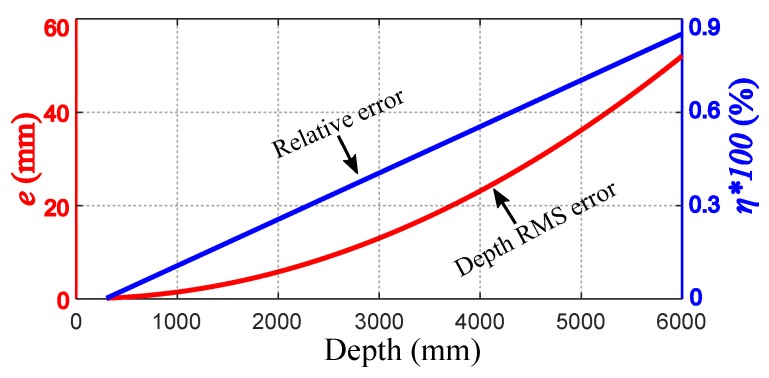
The depth RMS error and relative error.

The depth RMS error and relative error are shown in Figure 4. It can be seen that the absolute RMS error of the measured value is a quadratic function of the measured depth *z*, and the relative error has a linear relationship with the measured depth *z*.

### 5.2. Registration Results

**Figure 5:**
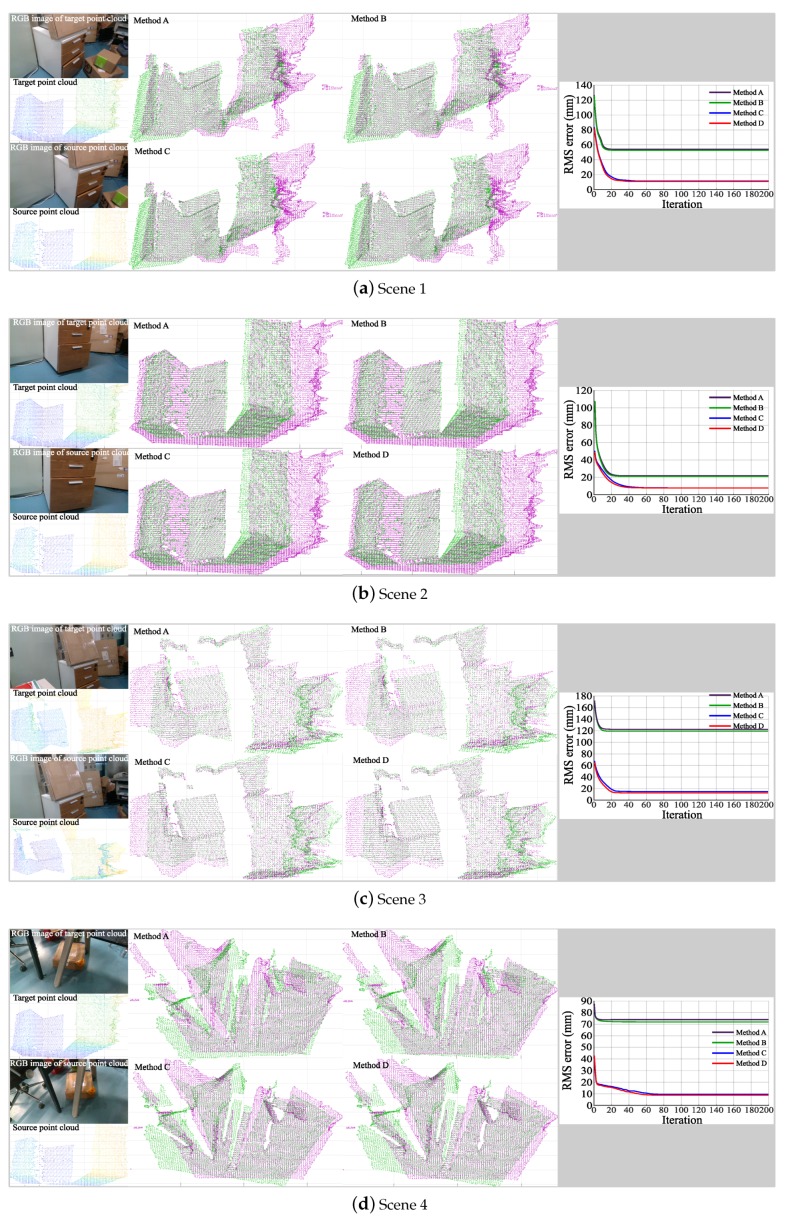
Comparison of point cloud registration results with different methods. **Method A**: original ICP algorithm, **Method B**: ICP algorithm with weighting, **Method C**: ICP algorithm with Q point pairs, **Method D**: ICP algorithm weighted by Q point pairs.

To verify the effectiveness of the proposed method, we complete the experiments of point cloud acquisition and registration in four different indoor scenes. In each scene, four different methods of registration are implemented: (A) original ICP algorithm; (B) ICP algorithm with weighting; (C) ICP algorithm with *Q* point pairs, (D) ICP algorithm weighted by *Q* point pairs, where *Q* is determined as 0.8 times the number of target point clouds. The experimental results are shown in Figure 5. To show the scene represented by point cloud, the RGB images of target and source point clouds are also presented. The registration RMS error in Method D is calculated by the square root of ϵ, where ϵ is calculated by Formula (19).

According to the registration results, the registration errors of the four methods in four different scenes are all convergent. Method A and Method B use all point pairs to calculate the rotation and translation matrices, but the weights of each pair of points between two methods are different. In Method A, the weights of all point pairs are equal, and in Method B, the greater the measurement error, the smaller the weight. It can be seen that Method B has smaller errors and higher registration accuracy which considers the effect of measurement errors than Method A. Similarly, when *Q* point pairs are used for registration, such as Method C and Method D, the registration results are also better when the measurement errors are taken into account. The results show that the measurement errors have an impact on the speed and accuracy of registration. When different numbers of point pairs are used for registration, the accuracy of registration is quite different. The registration results of Method C and Method D are obviously better than those of Method A and Method B. Therefore, the ICP algorithm weighted by *Q* point pairs are proposed in this paper, which can effectively improve the convergence speed of registration error and reduce the registration error.

## 6. Conclusions

Aiming at the influence of the number of registration points and measurement errors on the registration results, an improved ICP registration method is proposed in this paper. During the iterative registration process, only the point pairs with smaller Euclidean distances are used, which improves the accuracy of registration. To reduce the influence of measurement errors on registration result, the depth measurement error model based on Intel${}^{®}$ RealSense${}^{\mathrm{TM}}$ depth camera is analyzed, and an ICP registration method with error weights is constructed to improve the convergence speed of registration errors. The registration experiments in different indoor scenes demonstrate the effectiveness of the proposed improved ICP algorithm. The effects of measurement error and Euclidean distance of point pairs on registration result are studied in this paper. In future work, point cloud registration based on 4D modeling will be considered, which will make full use of the environmental features, and may improve the registration accuracy and speed [13,14,15].

## Figures and Tables

**Table 1:** The key specifications of Intel${}^{®}$ RealSense™ D415.

Item	Value
Depth technology	Active IR stereo
Depth Stream Output Resolution	1280 × 720
Depth Stream Output Frame Rate	30 fps
Minimum Depth Distance (Min-Z)	0.3 m
Maximum Range	Approx. 10 m
Depth Field of View (FOV)	69.4${}^{\circ }$ × 42.5${}^{\circ }$ × 77${}^{\circ }$ (±3${}^{\circ }$)
Baseline	55 mm
Subpixel	0.08
